# Development and Mechanical Characterization of Short Curauá Fiber-Reinforced PLA Composites Made via Fused Deposition Modeling

**DOI:** 10.3390/polym14225047

**Published:** 2022-11-21

**Authors:** Daniel K. K. Cavalcanti, Jorge S. S. Neto, Henrique F. M. de Queiroz, Yiyun. Wu, Victor F. S. Neto, Mariana D. Banea

**Affiliations:** 1 Federal Centre of Technological Education in Rio de Janeiro (CEFET/RJ), Rio de Janeiro 20271-110, Brazil; 2 TEMA-Centre for Mechanical Technology and Automation, Department of Mechanical Engineering, University of Aveiro, 3810-193 Aveiro, Portugal; 3 LASI-Intelligent Systems Associate Laboratory, 4800-058 Guimarães, Portugal; 4 CICECO-Aveiro Institute of Materials, Department of Materials and Ceramic Engineering, University of Aveiro, 3810-193 Aveiro, Portugal

**Keywords:** 3D printing, additive manufacturing, short fiber reinforcements, mechanical properties, PLA

## Abstract

The increase in the use of additive manufacturing (AM) has led to the need for filaments with specific and functional properties in face of requirements of structural parts production. The use of eco-friendly reinforcements (i.e., natural fibers) as an alternative to those more traditional synthetic counterparts is still scarce and requires further investigation. The main objective of this work was to develop short curauá fiber-reinforced polylactic acid (PLA) composites made via fused deposition modeling. Three different fiber lengths (3, 6, and 8 mm), and three concentrations in terms of weight percentage (2, 3.5, and 5 wt.%) were used to fabricate reinforced PLA filaments. Tensile and flexural tests in accordance with their respective American Society for Testing and Materials (ASTM) standards were performed. A thermal analysis was also carried out in order to investigate the thermal stability of the new materials. It was found that the main driving factor for the variation in mechanical properties was the fiber weight fraction. The increase in fiber length did not provide any significant benefit on the mechanical properties of the curauá fiber-reinforced PLA composite printed parts. The composites produced with PLA filaments reinforced by 3 mm 2% curauá fiber presented the overall best mechanical and thermal properties of all studied groups. The curauá fiber-reinforced PLA composites made via fused deposition modeling may be a promising innovation to improve the performance of these materials, which might enable them to serve for new applications.

## 1. Introduction

Additive manufacturing (AM), also known as 3D printing technology, is an innovative process in which parts can be manufactured with high precision and complexity. The feedstock used in the process is deposited layer by layer, and these materials are widely used in both household and industrial applications [1,2,3,4]. There are a wide variety of technologies used in 3D printing, where fused filament fabrication (FFF) or fused deposition modeling (FDM) is one of the most widely used AM techniques due to its ability to manufacture complex and relatively high-strength parts from low-cost materials [5,6,7]. The parts manufactured by FDM printers were found to be highly dependent on the feedstock material and processing parameters [8], as well as susceptible to printing defects (i.e., satellite droplets and nozzle clogging) [9,10]. The most common materials used in FDM are thermoplastics, among which polylactic acid (PLA) and acrylonitrile butadiene styrene (ABS) are the most common [11,12,13,14,15,16,17,18,19]. PLA is a linear aliphatic thermoplastic polyester, produced from renewable biodegradable materials, with good mechanical properties, thermal stability, processability, and low environmental impact [18]. The PLA presents many advantages over other thermoplastics used in FDM, including its lower melting temperature and reduced emissions of smells and plastic particles during printing. However, PLA still presents relatively lower mechanical properties compared to other plastics used in the industry. Therefore, to broaden the possible application of PLA printed parts numerous studies have focused on a wide range of reinforcing techniques [20]. The use of fiber reinforcements is common in composite materials, and a similar trend can be seen for the filaments used in FDM.

The use of continuous fiber-reinforced filaments has proven to be an effective method in increasing the mechanical properties of 3D printed composites. However, the necessity of specialized equipment to print and manufacture the filaments limits their applications, especially when printing parameters are concerned [21]. As an alternative, the use of short fibers as reinforcements for filaments can provide an increase in mechanical properties without the need of more complex fabrication and printing [22]. The reinforcements used to strengthen the filament can be either synthetic or natural [23,24,25]. However, the former presents problems for production due to higher abrasion and production cost when compared to natural fiber alternatives. Furthermore, natural fibers present advantages such as lower overall production costs, renewable source, and low abrasiveness [22,26,27]. However, the study of short natural fibers as reinforcements to thermoplastic filaments is scarce and can provide a possible alternative to synthetic reinforcement.

The main objective of this work was to develop and investigate the mechanical and thermal properties of 3D PLA printed parts reinforced by short natural (curauá) fiber. Three different fiber lengths (3, 6, and 8 mm) and three concentrations in terms of weight percentage (2, 3.5, and 5 wt.%) were used to fabricate reinforced PLA filaments. Tensile and flexural tests in accordance with their respective ASTM standards were performed. A thermal analysis was also carried out in order to investigate the thermal stability of the new materials.

## 2. Materials and Methods

### 2.1. Materials

The thermoplastic material used in this work was the PLA 4032D obtained as 6 mm pellets from Tucab (Leiria, Portugal). The reinforcement fibers were short curauá fibers, in natura by the Federal University of Pará (Universidade Federal do Pará, UFPA, Brazil). The mechanical properties of the materials used in this work are summarized in Table 1. The curauá is a type of natural fiber endemic to Brazil, they presented excellent mechanical properties, with high cellulose count and low lignin content (70.4% cellulose, 10.8% hemicellulose, and 11.1% lignin) [28]. The curauá fibers were manually cut, and their average size was measured through the Fiji image software [29]. The nomenclature used in this study can be seen in Table 2 (where C is curauá followed by average fiber length and fiber weight concentration).

### 2.2. Filament and 3D Part Specimens’ Fabrication

**Figure 1:**
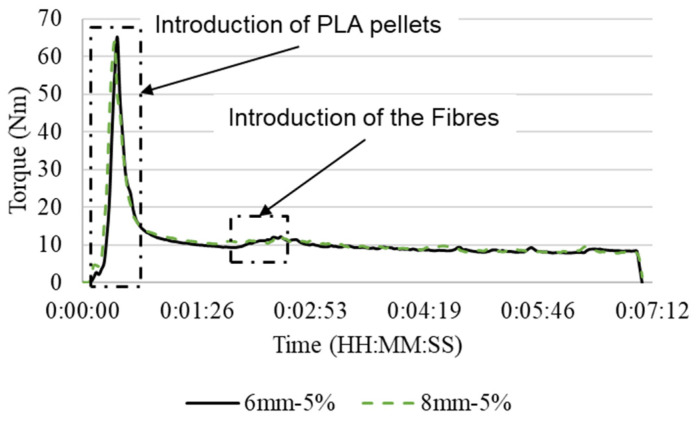
Representative Torque/time of the PLA–curauá mixing process.

The PLA–Curauá short fiber mixture was fabricated by means of a laboratory-scale mechanical melt-mixing Brabender^®^ Plastograph torque rheometer (BRABENDER GMBH & CO.KG, Duisburg, Germany). The PLA pellets and curauá fibers were mixed at a temperature of 180 °C and a speed of 100 rpm. The batches were separated in 25 g of PLA and the proportional wt.% in curauá fibers (i.e., 2%, 3.5%, and 5%). The natural fibers were added after the PLA presented stable mixing (i.e., when the force measured by the Brabander mixer begins to stabilize). In Figure 1, the representative curve of mixing force (Nm)/time (HH:MM:SS) is shown. After the fibers were added, they were left to mix for approximately 6 min. The speed and mixing time were chosen as to limit PLA degradation [32]. The temperatures used for mixing and extrusion were under the degradation onset for the curauá fibers [26].

**Figure 2:**
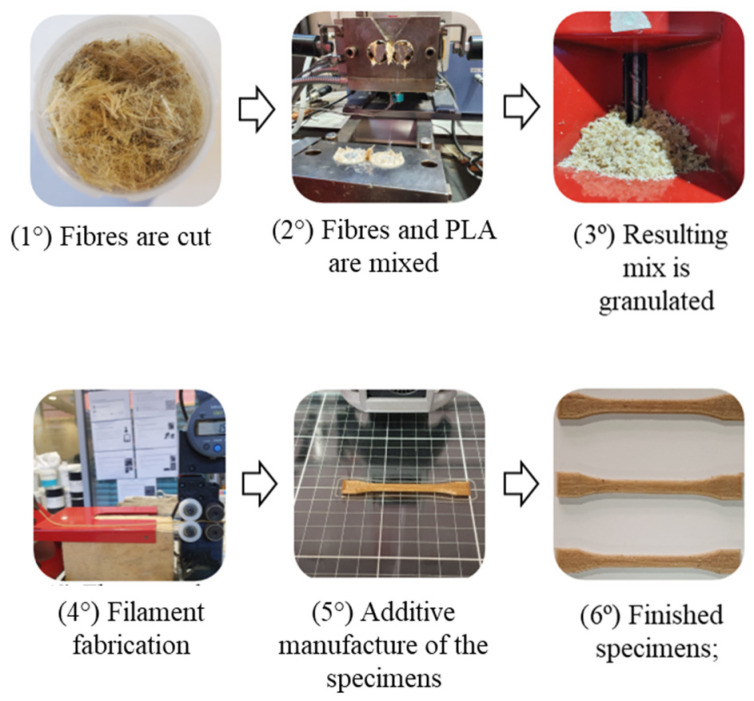
Schematic representation of the specimen fabrication.

**Figure 3:**
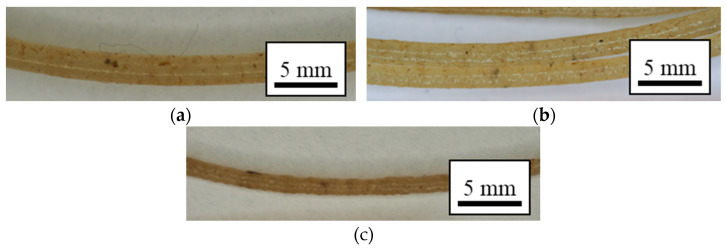
Representative images of the extruded filaments of the 3 mm group: (**a**) 2 wt.%, (**b**) 3.5 wt.%, and (**c**) 5 wt.%.

The resulting mixed composite was further ground in a Wanner Technik GmbH B08.10 (Wertheim-Reicholzheim, Germany) to a size of approximately 6 mm. These pellets were then fed in the single-screw Noztek pro filament extruder (Shoreham, England). The filaments with a variation in diameter of 1.5 ± 0.2 mm were manufactured with the aid of a Filafab (Wrington, England) puller and spooler machine. The processing schematics can be seen in Figure 2, and the extruded filament is shown in Figure 3.

**Figure 4:**
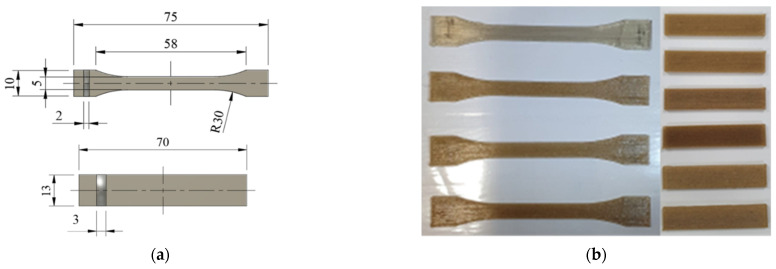
Tensile and flexural specimens: (**a**) schematic of the specimens with dimensions in millimeters (the specimen thickness is shown in gray) and (**b**) photos of the 3D printed specimens.

The 3D part specimens were fabricated using a B2X300 3D printer from BEEVERYCREATIVE (Ílhavo, Portugal) using the in-house manufactured PLA-Curauá filament. The standards upon which the specimens were produced were ISO 527 for the tensile tests and ASTM D790 for the flexural tests. The schematic representation of the specimens can be seen in Figure 4. At least five specimens were tested for each condition. 

The printing process parameters used in this work are presented in Table 3 and were chosen on the basis of the literature and in-house laboratory trials. The raster width chosen was 0.8 mm, above the normal value of 0.44 mm, which could be helpful to reduce internal voids produced by overlapping the rasters [7] while compensating for filament irregularity. The flow was increased (108%) in order to compensate for filament diameter variations (the increase in flow can help maintain pressure within the nozzle and help to reduce possible void and failures due to slippage and under extrusions). The raster angle chosen was 0°, as it was shown in the literature that a raster angle of 0° is the optimum direction of depositions if high tensile strength is desired. The infill chosen was 100%, due to the properties of the printed parts being associated with internal voids [8]. Slicing was performed with the Ultimaker Cura^®^ 4.12.1, and all test specimens were fabricated in a flat-edge orientation. The 0° infill direction was maintained by setting all layers as bottom layers in the specified angle. The production of 3D printed parts presents some drawbacks, especially when using noncommercial filaments, such as nozzle clogging [10]. In the present work these drawbacks were minimized by preliminary in-house tests by optimizing the print parameters. The filaments presented little clogging, with most printing defects originating from diameter inconsistencies.

### 2.3. Test Methods

#### Tensile and Flexural Tests

**Figure 5:**
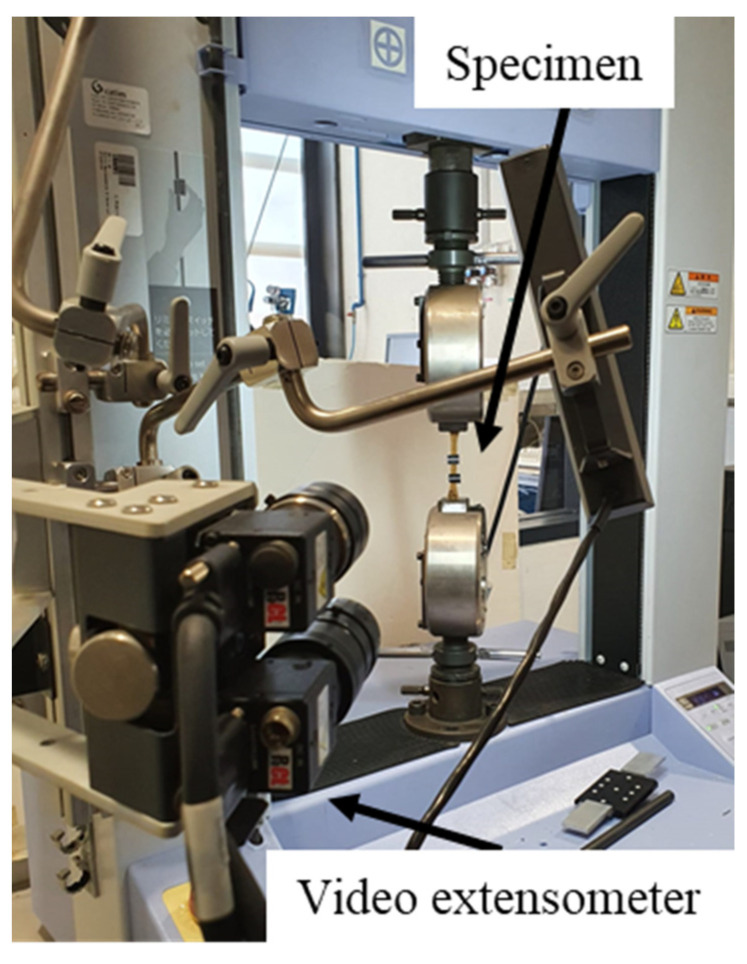
Tensile test setup.

The tensile and flexural tests were carried out in a SHIMADZU^®^ universal testing machine (Kyoto, Japan). The tensile tests were performed with a crosshead speed of 2 mm/min using a 10 kN load cell (see Figure 5). The specimen used for tensile tests followed the ISO 527-1:2019 size and shape. For the flexural tests, a three-point bending rig with a span of 120 mm and a cross-head speed of 1 mm/min, along with a 1 kN load cell, was used, as stated by the ASTM D790 standard. 

### 2.4. Thermal Analysis

#### 2.4.1. Thermogravimetric Analysis (TGA)

TGA was performed in a NETZSCH TG 209 F3 Tarsus machine (Netzsch-Gerätebau GmbH, Selb, Germany). Samples of approximately 20–30 mg were used to make the measurements. An alumina (Al_2_O_3_) crucible was used. Each sample was tested in the temperature range of 30–600 °C at a constant heating rate of 10 °C/min under nitrogen atmosphere (20 mL·min^−1^). 

#### 2.4.2. Differential Exploratory Calorimetry (DSC)

DSC was performed in a NETZSCH DSC 200 F3 Maia equipment (Netzsch-Gerätebau GmbH, Selb, Germany). The experiments were conducted with a heating rate of 10 K·min^−1^, in the temperature range of 30–250 °C with a nitrogen flux of 20 mL·min^−1^ according to ASTM D3418 with sample weights of approximately 8–10 mg. The glass transition temperature (T_g_), crystallization temperature (T_c_), and melting temperature (T_m_) were determined.

## 3. Results and Discussion

**Figure 6:**
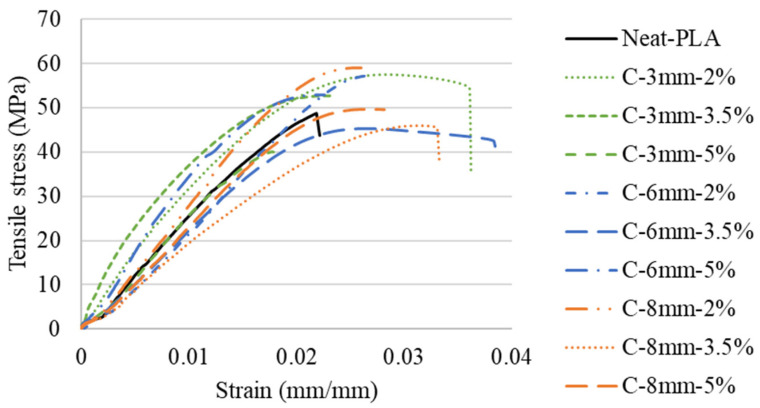
Representative tensile stress–strain curves.

**Figure 7:**
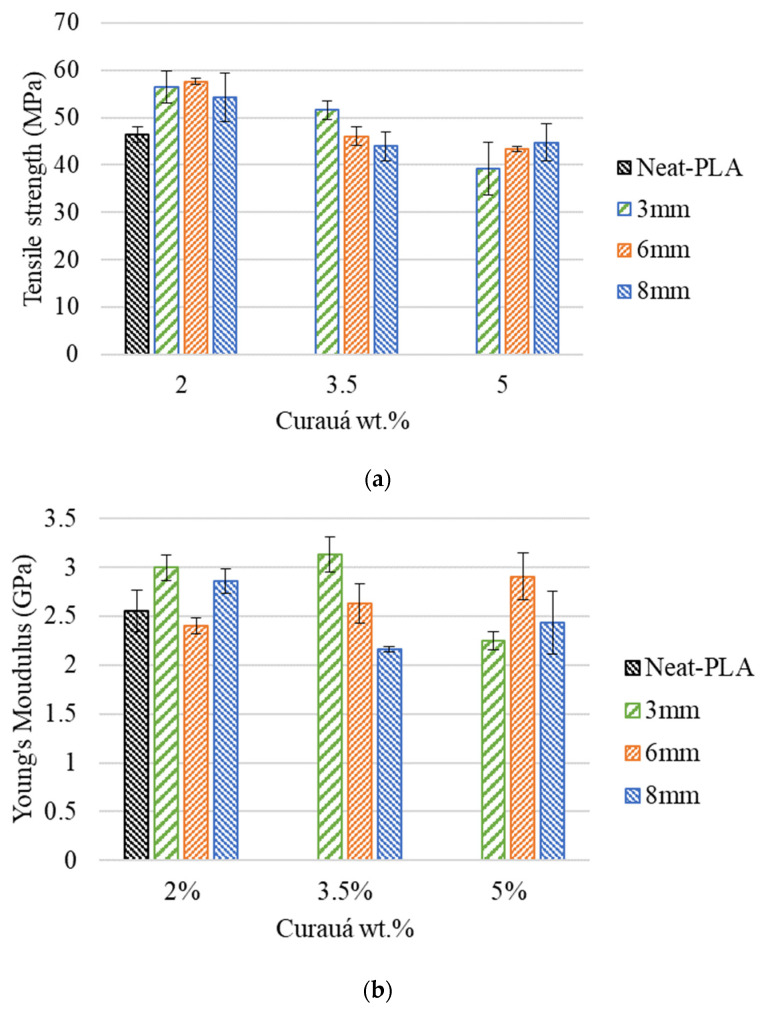
Tensile data of all composites studied: (**a**) tensile strength and (**b**) Young’s modulus.

The representative stress–strain curves obtained from the tensile tests as a function of reinforcement can be seen in Figure 6. The tensile data (tensile strength, Young’s modulus, and elongation at break) were compiled from the stress–strain curves, and the average results of the calculated data can be seen in Figure 7 and Table 4.

### 3.1. Effect of Curauá Fibre Weight Fraction

Fiber content had a positive effect on the 3D printed composite’s tensile strength when compared to the Neat-PLA. The C-3 mm-2%, C-6 mm-2%, and C-8 mm-2% cases presented increases of approximately 22%, 24%, and 17%, respectively, when compared to the Neat-PLA. Furthermore, an increase in tensile strength was also seen for the C-3 mm-3.5% (approximately 11%). This is mainly due to the curauá fiber’s higher tensile strength when compared to the PLA (Table 1) [22]. The increase in fiber content provided limited or no increase in tensile strength, mainly due to suboptimal fiber dispersion; thus, higher concentrations can act more as fillers (i.e., reducing the polymer content, potentially reducing costs with no significant gain in mechanical properties) than reinforcements [22,33].

The lower fiber wt.% presented the higher tensile strength. For example, the C-3 mm-2% group presented an increase of approximately 9% over the C-3 mm-3.5% and 44% over the C-3 mm-5% case. A similar behavior was seen for the 6 mm group, where the C-6 mm-2% case presented an increase of approximately 25% and 33% when compared to the C-6 mm-3.5% and C-6 mm-5% cases in tensile strength. Furthermore, the C-8 mm-2% group also presented higher tensile strength than the C-8 mm-3.5% and C-8 mm-5% specimens, with increases of approximately 24% and 21%, respectively. The results obtained reflect what is seen in the literature for natural fiber-reinforced filaments, where the increase in fiber content can reduce the tensile strength of the printed material [22,33]. This is mainly due to the saturation of fibers within the PLA during the mixing process (see Figure 4), resulting in a suboptimal mixture. Thus, the fibers begin to act more as a defect than a reinforcement. However, as seen in Berzin et al. [34], the interfacial interactions between fiber and matrix also play an important role in the specimen’s strength. As the PLA is a polar matrix [35], this would exert forces onto the curauá fibers, breaking bundles and single fibers, thus reducing their size and altering their aspect ratio. 

The Young’s modulus presented a similar behavior to the tensile strength, whereby an increase in rigidity was observed for most cases, when compared to the Neat-PLA. The exceptions were the C-6 mm-2%, C-6 mm-3.5%, and C-8 mm-5% cases, where no significant change was seen. Furthermore, decreases of approximately 12% and 15% in rigidity were observed for the C-3 mm-5% and C-8 mm-3.5% cases, respectively, when compared to the Neat-PLA. This is likely due to the same phenomenology as previously discussed for the tensile strength.

The strain at break of the reinforced specimens presented an increase when compared to the Neat-PLA. The 3 mm and 6 mm groups presented a downward tendency in strain at break with the increase in fiber content, with the C-3 mm-5% case presenting no significant difference from the Neat-PLA. However, no clear tendency was seen for the 8 mm group. The dispersion and values found for elongation can be explained by the variation of fiber property and dispersion within the printed specimen [36].

The inclusion of fiber treatments and different fabrication methods could be beneficial to the mitigation of fiber distribution and interfacial interaction problems. However, as seen in Pawłowska et al. [37], the utilization of such methods did not present clear benefits to the mechanical properties of the natural fiber-reinforced PLA. The differences in values found can be explained by the 3D printing process, which tends to better align the short fibers. Furthermore, the current work presented similar results to the work of Jesus et al. [38], where the inclusion of fibers had a positive impact on mechanical properties but only at a lower fiber wt.% content (0.5 wt.%). The increase in fiber wt.% did not provide any significant improvement in mechanical properties due to fiber agglomerations acting as stress concentrators.

### 3.2. Effect of Curauá Fiber Length

From Figure 7 and Table 4, it can be seen that the increase in fiber length had little impact on the mechanical properties (tensile strength and Young’s modulus). The tensile strength of the C-3 mm-2% case presented no significant variation from both the C-6 mm-2% and C-8 mm-2% specimens. Furthermore, the C-3 mm-3.5% case presented an overall higher tensile strength over both the C-6 mm-3.5% and C-8 mm-3.5% cases, having increases of approximately 12% and 17%, respectively. However, the C-3 mm-5% case presented a decrease in tensile strength, with reductions of approximately 10% and 12% when compared to the C-6 mm-5% and C-8 mm-5% cases, respectively.

Fiber length also had an impact in the stiffness properties of the materials. The C-3 mm-2% group presented an increase of approximately 33% over the C-6 mm-2% case, while, for the C-8 mm-2% case, no significant change in Young’s modulus was observed. The C-3 mm-3.5% case presented an improvement of approximately 10% and 45% over the C-6 mm-3.5% and C-8 mm-3.5% cases, respectively. However, with the increase in fiber wt.%, the C-3 mm-5% groups presented lower stiffness than the C-6 mm-5% case, presenting a reduction of approximately 23%. The C-8 mm-5% case did not present any significant statistical variation when compared to the C-3 mm-5% case. 

The data shows that, although fiber length had an impact in the tensile properties of the 3D printed specimens, it was not as relevant as the fiber content. As seen in Figure 7, the 2 wt.% curauá presented overall better tensile properties than the 3.5 or 5 wt.% curauá cases, regardless of fiber length. This is mainly due to the curauá fiber mixing process and inherent fiber characteristics. In the relevant literature, the diameter of the fibers used was much lower than that of the curauá fibers used in this study [22,23,24,39]. This increase in fiber diameter can hamper the mixing of higher fiber contents. Of note is also the interaction of the fiber with the matrix, as natural fibers have notoriously lower interfacial adhesion with most thermoplastic and thermoset matrices. However, the increase in fiber length and diameter can have a positive impact in tensile properties [22]. The combination of better fiber/matrix interfacial bond strength and a high interfacial surface area can be beneficial to the composite, presenting an increase in tensile strength, modulus, and elongation.

It was observed that the best overall mechanical tensile properties were found for the C-3 mm-2% case. Although, the 2% wt.% group presented similar results in tensile strength, the C-3 mm-2% case presented a higher Young’s modulus than the C-6 mm-2% case and similar results to the C-8 mm-2% case. As stated above, this was most likely due to better fiber distribution within the 3D printed specimens.

### 3.3. Flexural Tests

**Figure 8:**
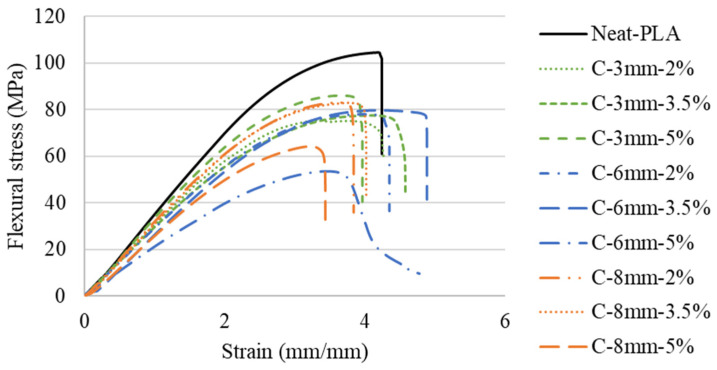
Representative flexural stress–strain curves.

**Figure 9:**
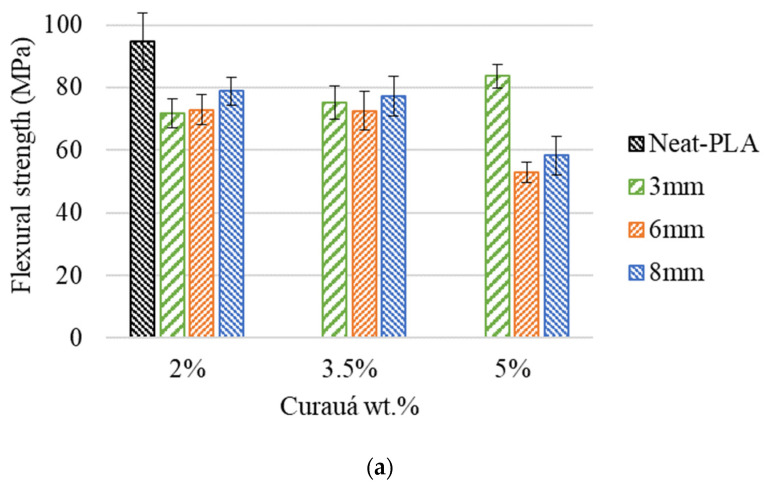

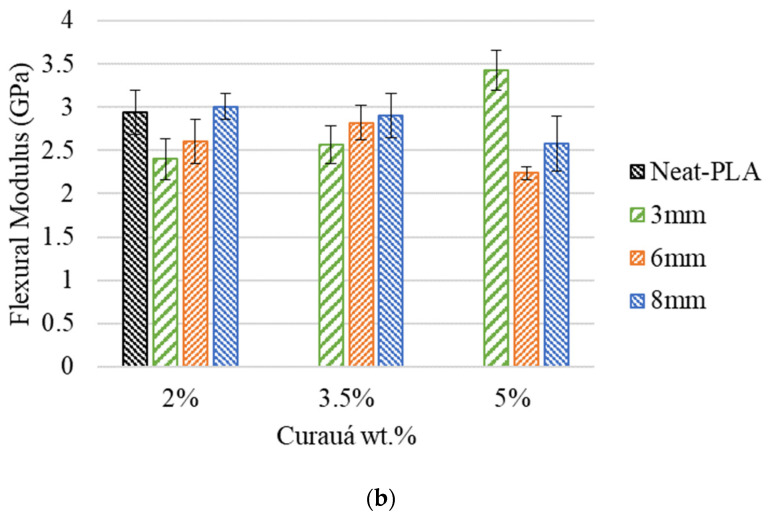
Flexural data of all composites as a function of fiber length and wt.%: (**a**) flexural strength and (**b**) flexural modulus.

The representative stress–strain curves obtained from the flexural tests as a function of fiber length and fiber wt.% can be seen in Figure 8. The flexural properties (strength and modulus) were calculated from the stress–strain curves, and the results are presented in Figure 9.

In general, the addition of natural fibers did not present any significant improvement in flexural strength (see Figure 9). The best result seen for the flexural strength of the fiber-reinforced specimens was the C-3 mm-5% group with a decrease of approximately 11% when compared to the Neat-PLA group. Within the reinforced groups, it is possible to see that the C-3 mm-5% case presented the best overall results, with increases of approximately 17% and 11% over the C-3 mm-2% and C-3 mm-3.5% groups. The 6 mm group presented a different trend, with the increase in fiber content providing a negative effect; both C-6 mm-2% and C-6 mm-3.5% cases presented an increase of approximately 38% when compared to the C-6 mm-5% case. The C-8 mm group continued the trend, presenting lower values with the increase in fiber wt.%. The C-8 mm-5% presented approximately 35% and 32% lower flexural strength when compared to C-8 mm-2% and C-8 mm-3.5%, respectively. No significant variation between C-8 mm-2% and C-8 mm-3.5% was seen. 

The flexural modulus followed a similar trend to the flexural strength. The flexural modulus presented no significant variation between the C-3 mm-2% and C-3 mm-3.5% cases, while the C-3 mm-5% case also presented an increase of approximately 43% when compared to the C-3 mm-2%. As seen in the flexural strength, C-6 mm-2% and C-6 mm-3.5% also did not present a significant change in stiffness. Following a similar behavior to the flexural strength, the lowest value was C-6 mm-5%, having reductions of approximately 14% and 21% when compared to the C-6 mm-2% and C-6 mm-3.5%, respectively. The 8 mm cases presented similar trends to the C-6 mm cases. Whereas C-8 mm-2% and C-8 mm-3.5% presented higher values than C-8 mm-5% (by approximately 14% and 11%, respectively), there was no significant variation between them. 

As seen in the tensile tests, the fiber weight fraction had a higher impact on flexural strength than fiber length. This is evident by the insignificant variation among the C-3 mm-2%, C-6 mm-2%, and C-8 mm-2% groups. Furthermore, C-3 mm-3.5%, C-6 mm-3.5%, and C-8 mm-3.5% presented similar behavior, with no significant variation between them. However, the C-3 mm-5%, C-6 mm-5%, and C-8 mm-5% groups presented significant variation, in which the C-3 mm-5% group presented approximately 58% and 44% increases in flexural strength. 

The flexural properties of the materials did not follow the trends set by the tensile tests. The flexural strength and modulus were affected negatively by the introduction of the fibers, apart from the C-3 mm-5% case, which presented an increase in stiffness of approximately 17% over the Neat-PLA. 

It has been shown in the literature that the addition of natural fibers as reinforcements for additive manufactured parts does not translate into an increase in flexural properties. Data presented a general trend of flexural strength reduction [22]. The present work followed this trend for most cases, with a reduction in flexural strength and modulus, with the only exception being the C-3 mm-5% group.

### 3.4. Fracture Analysis

**Figure 10:**
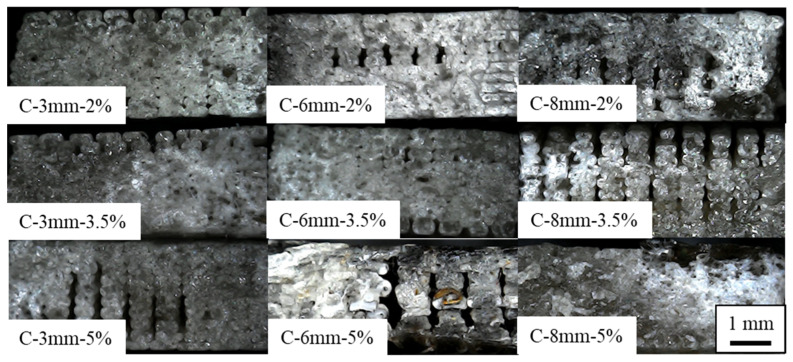
Representative failure modes of the 3D printed tensile specimens.

**Figure 11:**
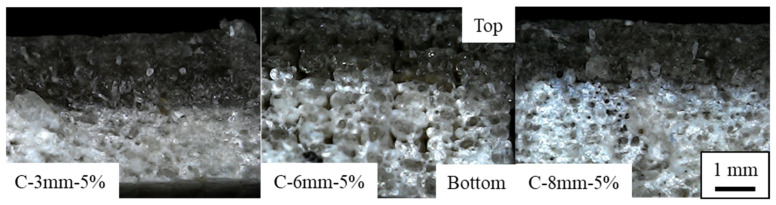
Representative failure modes of the 3D printed flexural specimens.

The fractured specimens were visually examined after the tensile and flexural tests. The representative failure modes can be seen in Figure 10 and Figure 11. The 3D printed composites presented a similar failure behavior. As seen in Figure 10, the tensile specimen failure was predominantly fragile with a clear stock break. The voids seen in the image are related to the separation between rasters. Although good interlayer adhesion can be seen, the failure tended to propagate between rasters as seen in Figure 10 for the C-3 mm-5%, C-6 mm-5%, and C-8 mm-3.5% cases. These defects can be attributed to the filament diameter variations, leading to a reduction in flow and resulting in a smaller raster width, thus hampering the interfacial interaction between them [40].

The representative flexural failure mode cross-sections can be seen in Figure 11. All failures presented a similar behavior with complete break; therefore, only the 5% group is shown. The failure surface presented two distinct areas separated approximately in the middle (neutral line). The bottom side (submitted to tensile loads) presented a rougher surface with less flat surfaces, which is likely due to deformation during the flexural tests. The top side (compressive side) presented a clearer failure, which is in line with a catastrophic failure with little to no deformation resulting in a pure fragile failure mode. 

## 4. Thermal Properties

### 4.1. TGA Analysis

**Figure 12:**
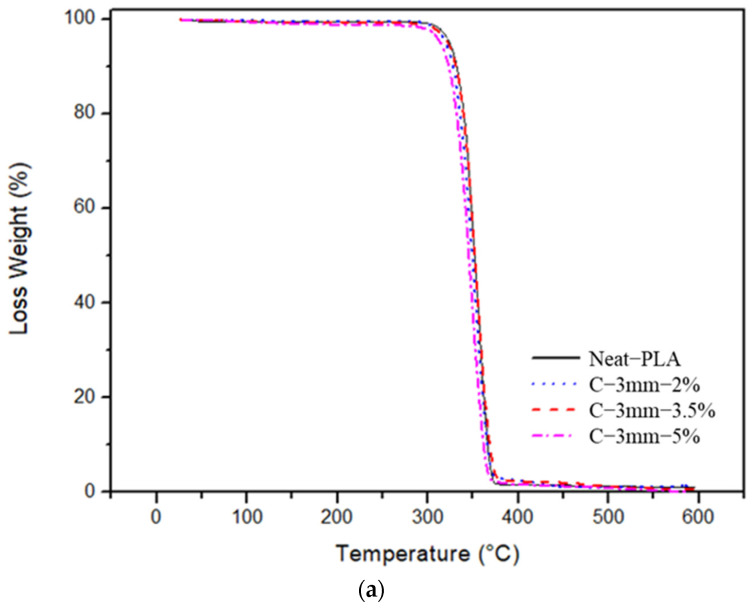

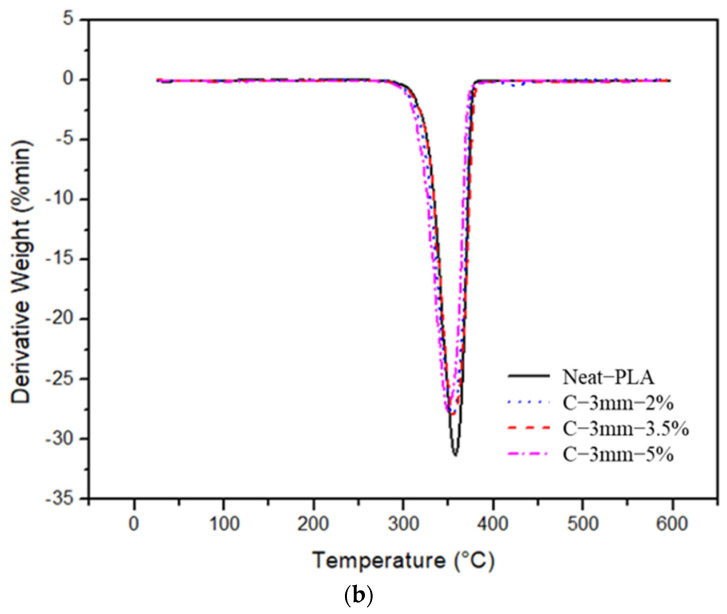
TGA analysis data: (**a**) TG curves; (**b**) DTG curves.

**Figure 13:**
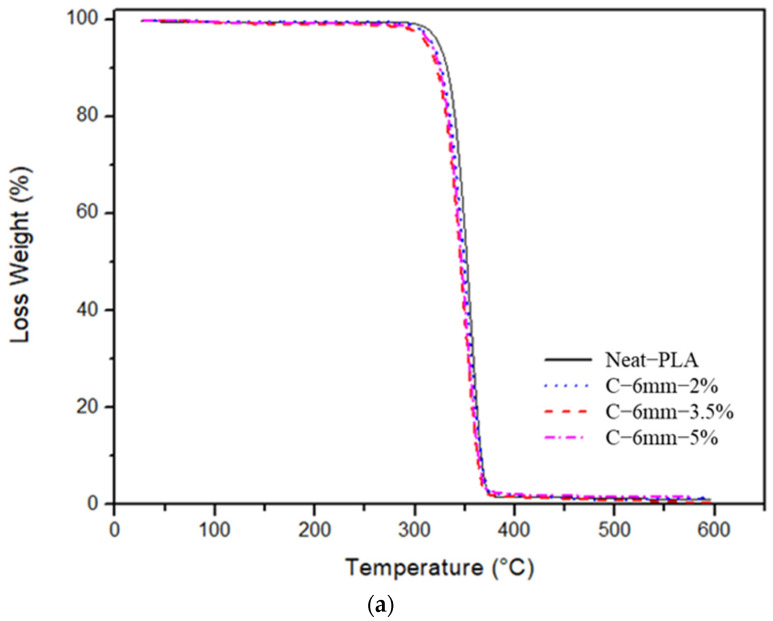

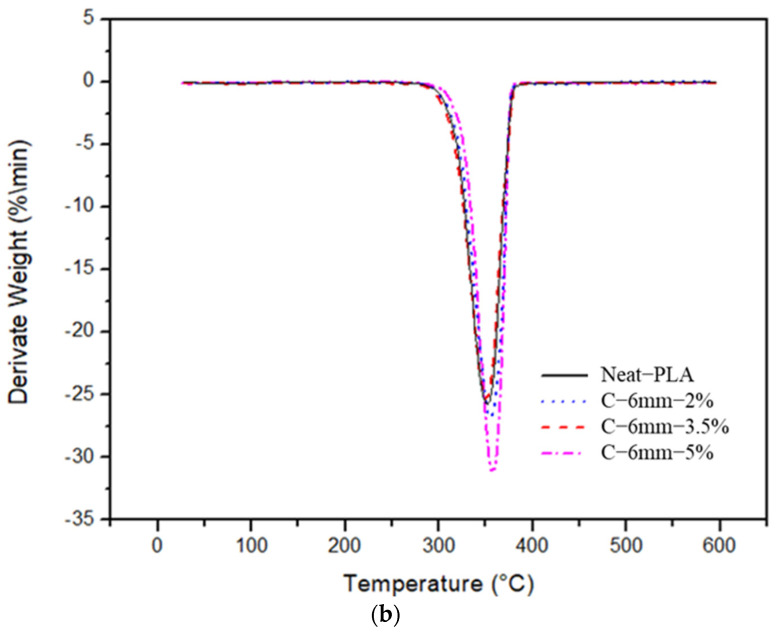
TGA analysis data: (**a**) TG curves; (**b**) DTG curves.

The thermal stability of curauá fiber-reinforced composite was investigated for two fiber (3 mm and 6 mm) length specimens and Neat-PLA. The weight loss and derivative weight are shown in Figure 12 and Figure 13. The Neat-PLA presented a temperature onset of 329.7 °C; throughout the whole heating process, only one degradation step was seen. The incorporation of the short fibers, as well as their size and concentration, had an impact in the composite’s thermal properties. The C-3 mm-2% case did not show significant changes in thermal properties (330 °C). The C-3 mm-3.5% case presented an increase in the onset temperature of 334 °C. However, the increase in fiber concentration for the C-3 mm-5% presented a reduction in temperature onset when compared to the Neat-PLA. The increase in fiber concentration reduced the thermal stability of the composites, mainly due to the inclusion of materials with lower thermal stability, such as such as hemicellulose and lignin [39]. An important factor in improving the thermal stability of natural fibers is the removal of these low-stability components through the use of chemical treatments [26,41]. Furthermore, C-3 mm-5% DTG (349 °C) presented a decay when compared to the Neat-PLA (357 °C), as seen in Figure 13b. 

From Table 5, it can be seen that the C-6 mm-2% and C-6 mm-5% specimens did not present any significant onset temperature variation when compared to the Neat-PLA specimens. However, for the C-6 mm-3.5% specimens, a deterioration of onset temperature (326 °C) when compared to the Neat-PLA was observed. As stated by Silva et al. [39], the incorporation of natural fibers had a tendency to reduce the thermal stability of composites in general, since these reinforcing natural fibers present lower thermal stability than the matrices.

### 4.2. DSC Analysis

**Figure 14:**
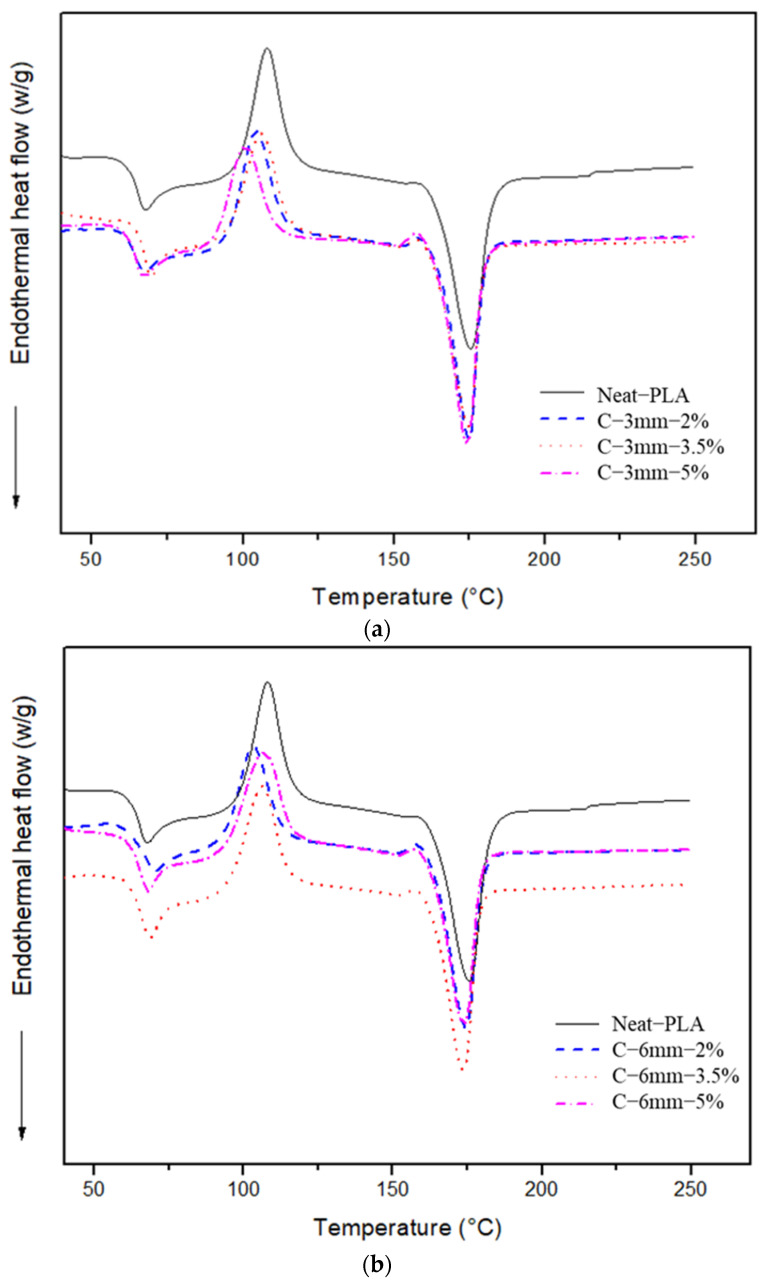
DSC analysis data: (**a**) 3 mm fiber length group; (**b**) 6 mm group and Neat-PLA.

DSC analysis was used to determine the following parameters: glass transition (T_g_), crystallization temperature (T_c_), and melting temperature (T_m_) of the specimens studied. The DSC curves produced are shown in Figure 14a,b. During the heating cycle, the materials showed three transition zones: glass transition at 60 to 68 °C [42], cold crystallization at 100 to 110 °C [43], and melting at 170 to 176 °C [44]. The Neat-PLA presented a T_g_ value of 64.5 °C (see Table 6). The value found is in accordance with the literature [45,46]. For the reinforced (2%, 3.5%, and 5%) composites, a change in the thermal properties with the insertion of the curauá fiber in the composite was observed (a small increase in T_g_ for the 2 and 3.5 wt.% when compared to Neat-PLA was observed). However, the case of C-3 mm-5% showed no significant change in the T_g_ of the material. The case of composite C-6 mm-2% showed an increase in T_g_ (1.6 °C) when compared to Neat-PLA. However, C-6 mm-3.5% and C-6 mm-5% showed no significant change in T_g_. It was shown in the literature that short natural fibers are well dispersed in the matrix, where they can act in the nucleation zones of the polymer crystallization or in the restriction of the polymeric chains [45]. As for the crystallization temperature, all cases showed a decrease when compared to the Neat-PLA (see Table 6). Xia et al. [47] also found that the incorporation of natural fibers decreased the T_c_ when compared to Neat-PLA. 

The 6 mm fiber length specimens presented a decrease in T_c_ compared to Neat-PLA, exhibiting the same behavior as the 3 mm length fiber-reinforced composites. For the two cases of C-3 mm-(3.5 and 5 wt.%) and 6 mm-(2, 3.5, and 5 wt.%) fiber length specimens, a small decrease in Tm was observed when compared to Neat-PLA. Akonda et al. [48], showed that the flax/PLA composite presented a decrease in Tm at the beginning of the melting temperature of the composites. However, the C-3 mm-2% specimens showed no significant change in Tm. These results are in accordance with results found in the literature [49]. 

## 5. Conclusions

In this work, short curauá fiber-reinforced PLA composites made via fused deposition modeling were investigated. Three different fiber lengths (3, 6, and 8 mm) and three concentrations in terms of fiber weight percentage (2, 3.5, and 5 wt.%) were used to fabricate the PLA filaments. Tensile and flexural tests in accordance with their respective ASTM standards were performed. A thermal analysis was also carried out in order to investigate the thermal stability of the new materials. In general, the addition of the short fibers increased the tensile properties of the 3D printed specimens, with both tensile strength and Young’s modulus benefiting from the reinforcements. The following conclusions can be drawn:

- The fiber weight fraction dominated the tensile properties of the 3D printed parts, with lower content (2 wt.%) presenting a significant improvement in tensile properties. Fiber length also affected the tensile properties; however, a downward trend was seen with the increase in length, with the C-3 mm-2% (56.45 MPa and 3 GPa) group presenting the best results.
- For flexural properties, the best condition was the C-3 mm-5% case, with values of 83.71 MPa and 3.42 Gpa for flexural strength and modulus, respectively. Unlike the tensile properties, the addition of the fibers did not provide an increase in flexural strength, with the best case presenting a reduction of 12% when compared to the Neat-PLA. However, the reinforcements did have a positive impact on the flexural modulus (the C-3 mm-5% specimens presented an increase of approximately 17% over the Neat-PLA).
- The incorporation of natural fiber presented, in some cases, changes in the composite’s thermal stability. The C-3 mm-3.5% specimens presented the best condition with values of 334 °C for Tonset and 67.5 °C for T_g_.

Adding curauá short fibers to PLA filaments for 3D printed materials may be a promising innovation to improve their mechanical and thermal properties while maintaining their biodegradable characteristics. The present work adds to the ever-growing green industry, with possible applications in biomedical, food packing, disposable single-use components, eco-friendly molds, and lightweight customizable parts for the transport industry. Further studies on the use of this type of fiber are necessary to prove that natural fibers are a good substitute to more commonly used synthetic fibers.

## Figures and Tables

**Table 1:** Material properties.

Material	Tensile Strength (MPa)	Young’s Modulus (GPa)	Elongation at Break (%)	Density (g/cm^3^)
PLA 4032D [30]	51.00 ± 5.00	3.08 ± 0.30	5.76 ± 0.50	1.24
Curauá [31]	1929.80 ± 249.50	87.23 ± 15.40	3.94 ± 0.60	1.33

**Table 2:** Nomenclature of the specimens as a function of reinforcement (average fiber length and wt.%).

Specimen Type	Fibre Length (mm)	Weight Concentration (wt.%)
Neat-PLA	-	-
C-3 mm-2%	3.02 ± 0.97	2
C-3 mm-3.5%		3.5
C-3 mm-5%		5
C-6 mm-2%	6.53 ± 0.99	2
C-6 mm-3.5%		3.5
C-6 mm-5%		5
C-8 mm-2%	7.97 ± 0.92	2
C-8 mm-3.5%		3.5
C-8 mm-5%		5

**Table 3:** The printing parameters used for the 3D printed specimens.

Printing Parameters	Value
Nozzle diameter (mm)	0.40
Layer height (mm)	0.20
Raster width (mm)	0.8
Raster angle	0°
Infill %	100
Extruder temperature (°C)	220
Printing bed temperature (°C)	50
Printing speed (mm/s)	30
Number of contours	1
Flow (%)	108

**Table 4:** Tensile data of all composites studied.

Part	Tensile Strength (MPa)	Young’s Modulus (GPa)	Strain (%)
Neat-PLA	46.42 ± 1.62	2.56 ± 0.21	1.94 ± 0.15
C-3 mm-2%	56.45 ± 3.34	3.00 ± 0.13	2.72 ± 0.31
C-3 mm-3.5%	51.58 ± 1.9	3.13 ± 0.08	2.32 ± 0.38
C-3 mm-5%	39.15 ± 5.55	2.25 ± 0.12	1.81 ± 0.68
C-6 mm-2%	57.56 ± 0.67	2.4 ± 0.18	2.59 ± 0.18
C-6 mm-3.5%	45.98 ± 1.95	2.63 ± 0.2	2.51 ± 0.11
C-6 mm-5%	43.29 ± 0.56	2.91 ± 0.02	2.21 ± 0.1
C-8 mm-2%	54.25 ± 5.22	2.86 ± 0.09	2.34 ± 0.27
C-8 mm-3.5%	43.91 ± 3.02	2.16 ± 0.24	3.01 ± 0.46
C-8 mm-5%	44.76 ± 3.96	2.43 ± 0.32	2.62 ± 0.23

**Table 5:** TGA analysis data.

Specimen Type	Onset Temperature (°C)	Maximum Temperature (°C)	Residual Mass (%)
Neat-PLA	330	357.5	1
C-3 mm-2%	330.0	355.0	1.4
C-3 mm-3.5%	334.0	354.5	0.7
C-3 mm-5%	327.8	349.0	0.2
C-6 mm-2%	329.6	355.0	1.4
C-6 mm-3.5%	326.3	350.0	0.4
C-6 mm-5%	328.0	351.5	1.6

**Table 6:** DSC analysis data.

Specimen Type	Glass Transition Temperatures (T_g_)	Crystallization Temperatures (T_c_)	Melting Temperature (T_m_)
Neat-PLA	64.0	108.1	175.5
C-3 mm-2%	65.1	104.1	175.0
C-3 mm-3.5%	67.5	106.4	174.7
C-3 mm-5%	64.3	100.9	174.2
C-6 mm-2%	66.1	103.4	174.3
C-6 mm-3.5%	64.0	106.3	173.5
C-6 mm-5%	64.4	106.5	174.2

## Data Availability

Not applicable.
